# CD36 in asthma: from a candidate immunometabolic checkpoint to genetic susceptibility

**DOI:** 10.3389/fimmu.2026.1924772

**Published:** 2026-09-01

**Authors:** Xingjie Li, Chumeng Zhang, Shanshan Zhao, Xiao Chang, Hua Tang

**Affiliations:** 1 Shandong Provincial Lab for Clinical Immunology Translational Medicine in Universities, The First Affiliated Hospital of Shandong First Medical University and Shandong Provincial Qianfoshan Hospital, Jinan, Shandong, China; 2 Institute of Infection and Immunity, Medical Science and Technology Innovation Center, Shandong First Medical University and Shandong Academy of Medical Sciences, Jinan, Shandong, China; 3 School of Clinical and Basic Medicine, Shandong First Medical University, Jinan, Shandong, China; 4 Department of Clinical Laboratory, Guangyuan Central Hospital, Guangyuan, Sichuan, China; 5 College of Medical Information and Artificial Intelligence, Shandong First Medical University, Jinan, Shandong, China; 6 Department of Otorhinolaryngology, Head and Neck Surgery, Yantai Yuhuangding Hospital, Qingdao University, Yantai, Shandong, China

**Keywords:** asthma, CD36, immunometabolic checkpoint, PPAR-γ, type 2 inflammation

## Abstract

Asthma arises from complex interactions between genetic susceptibility and environmental exposure, yet the molecular pathways linking these processes remain incompletely understood. Recent human genetic studies have implicated CD36, a multifunctional class B scavenger receptor, in asthma risk, renewing interest in its immunological functions beyond lipid transport. Accumulating evidence suggests that CD36 influences multiple stages of disease pathogenesis, including allergen recognition and uptake, immune-cell activation and lipid-dependent metabolic adaptation. The identification of the asthma-protective variant rs75326924 has further connected CD36 biology to human disease and provided a genetic framework for mechanistic investigation. At the same time, emerging studies suggest that CD36 integrates extracellular lipid cues with intracellular immunometabolic programs that support persistent inflammatory responses, thereby providing a potential mechanistic link between environmental exposure and disease susceptibility. In this review, we discuss current evidence linking CD36 to asthma susceptibility and pathogenesis, evaluate the mechanistic basis of its immunological activities and discuss its potential as a biomarker and therapeutic target. We propose that CD36 represents a candidate immunometabolic checkpoint at the intersection of environmental sensing, metabolic regulation and genetic risk.

## 1 Introduction

### 1.1 Asthma: persistent clinical challenges

Despite significant advances in the diagnosis and treatment of asthma, more than 300 million people worldwide still suffer from the condition (1, 2). Asthma is a biologically heterogeneous syndrome comprising a variety of conditions, primarily characterized by chronic airway inflammation, airway hyperresponsiveness, and variable airflow limitation (3).

Among the recognized inflammatory endotypes of asthma, type 2 (T2) inflammation is the most prevalent. It is characterized by eosinophilic airway inflammation together with activation of T helper 2 (Th2) cells and group 2 innate lymphoid cells (ILC2s), and accounts for approximately half of all asthma cases. In contrast, T2-low asthma encompasses neutrophilic and paucigranulocytic endotypes with more heterogeneous mechanisms, including Th1/Th17-associated inflammation, oxidative stress, and neuroimmune pathways (4, 5). Currently, the development of biologics targeting IgE, IL-5, IL-4Rα, and thymic stromal lymphopoietin (TSLP) has significantly transformed the treatment landscape for severe T2 asthma (6, 7). Outcomes show substantial suppression of downstream inflammatory mediators, but a substantial proportion of patients continue to experience acute exacerbations, persistent symptoms, or progressive deterioration in lung function (8).

Asthma progression is driven by environmental exposure, epithelial alarm signals, immunometabolism and genetic background, and blocking any single inflammatory pathway alone will not provide sufficient benefit. Hence, it is necessary to find molecular nodes that can integrate these layers.

### 1.2 Immunometabolism as an emerging framework in asthma

Over the past decade, immunometabolism has fundamentally transformed our understanding of immune cell biology. Metabolic pathways are now recognized not only as sources of cellular energy but also as active regulators of immune cell differentiation, survival, and effector functions (9). In asthma, a growing body of evidence suggests that lipid metabolism is directly involved in the pathogenesis of the disease. Fatty acid uptake, mitochondrial β-oxidation, and lipid-mediated signaling influence the activation of various immune cells, including macrophages, dendritic cells, eosinophils, and ILC2s (10–12). Consequently, molecules that integrate metabolic and inflammatory signals may occupy a pivotal position in the disease network. Among these candidate molecules, CD36 is of particular interest.

### 1.3 CD36: beyond a lipid transporter

CD36 is a multifunctional class B scavenger receptor that is widely expressed in various immune and parenchymal cells, including macrophages, dendritic cells, platelets, endothelial cells, epithelial cells, and ILC2s (13, 14). It has been studied primarily in the context of atherosclerosis and metabolic diseases due to its ability to mediate the uptake of long-chain fatty acids and oxidized lipoproteins.

However, CD36 also functions similarly to pattern recognition receptors, interacting with oxidized phospholipids, apoptotic cells, microbial products, and endogenous danger-associated molecular patterns (15). Through synergistic interactions with signaling pathways such as Src family kinases, Toll-like receptors (TLRs), and MAPK cascades, CD36 influences inflammatory responses that extend far beyond the scope of lipid metabolism (16–18).

Recent studies have further demonstrated that CD36 participates in the metabolic programming of immune cells, particularly those associated with T2 immunity. In activated ILC2s, CD36-dependent fatty acid uptake contributes to mitochondrial metabolism and cytokine production, revealing a previously underappreciated link between nutrient sensing and allergic inflammation (19).

### 1.4 Human genetics revives interest in CD36

Interest in CD36 as a candidate asthma susceptibility gene has been stimulated by recent advances in human genetics. A genome-wide association study (GWAS) identified the East Asian-enriched missense variant rs75326924 as a protective allele associated with reduced asthma susceptibility and lower eosinophil counts. Unlike many candidate genes supported primarily by differential expression studies, CD36 is therefore linked to asthma by direct human genetic evidence (20). This finding has renewed interest in the biological functions of CD36 and raises the possibility that it contributes to asthma pathogenesis through mechanisms extending beyond lipid metabolism. The genetic and mechanistic evidence supporting this concept is discussed in detail below.

## 2 Why CD36 emerged as an asthma susceptibility gene

### 2.1 From lipid biology to asthma genetics

CD36 has been extensively studied in lipid metabolism and atherosclerosis, while human genetic studies have also linked CD36 variation to metabolic syndrome and related lipid traits (21, 22). Its ability to mediate the uptake of long-chain fatty acids and oxidized lipids has established CD36’s key role in the regulation of cellular energy homeostasis. However, the receptor’s broad ligand repertoire and its widespread expression in various immune cells suggest that its functions may extend beyond the metabolic sphere.

Interest in asthma stems from experimental observations showing elevated CD36 expression in the airways and peripheral immune cells of asthma patients (20). Increased CD36 expression is associated with eosinophilic inflammation and disease severity, while CD36-deficient mice exhibit reduced airway eosinophil infiltration, decreased production of IL-4, IL-5 and IL-13, and attenuated airway hyperresponsiveness in a house dust mite-induced asthma model (23, 24). Although these findings are insightful, it remains unclear whether CD36 is a driver of the disease or merely reflects the ongoing inflammatory process.

### 2.2 Human genetic and molecular evidence supports disease relevance

The rs75326924 variant is a rare missense mutation in the CD36 gene that is associated with a reduced risk of asthma in East Asian populations. This association was further supported in a Chinese cohort study, in which the variant demonstrated a consistent protective effect. The association showed a similar direction of effect in an independent Korean cohort, although it did not reach statistical significance (Beta = −0.54, *P* = 0.086) (20, 25).

Notably, human molecular and cellular-level data provide additional evidence for the role of CD36 in asthma. RNA-sequencing analysis of primary bronchial epithelial cells from 88 asthma patients and 42 healthy controls revealed elevated CD36 expression in asthma patients. This indicates that CD36 is located at the airway epithelial interface, a key site of exposure to environmental factors. In peripheral blood, flow cytometry analysis further demonstrated increased CD36 expression in lymphocytes from asthma patients. CD4^−^CRTH2^+^ cells, which define the ILC2-rich population, also exhibited higher levels of CD36 expression in asthma patients. These findings provide evidence at the human cellular level that CD36-related changes are widespread in the airways and immune system associated with allergic inflammation.

The same study combined genotyping with plasma proteomics to investigate the molecular phenotype associated with the rs75326924 locus. In the Olink inflammatory protein assay panel—which included 1,056 Chinese individuals and covered 365 proteins—individuals carrying the variant allele exhibited differential expression of 43 proteins, with the most significantly affected immune-related proteins including IL-7, OSM, and VEGFA. These results suggest that genetic variants in CD36 may be associated with broader alterations in inflammatory regulation. However, only five individuals in this proteomic cohort carried this variant; therefore, the observed protein differences require independent validation and cannot be interpreted as direct downstream effects of CD36 dysfunction. Population genetic analysis provides additional context regarding the distribution of rs75326924. This locus exhibits significant population differentiation in East Asian populations, with high Fst and iHS values consistent with the evolutionary history of specific populations (20). This population specificity may be particularly important for future research using CD36 as a biomarker or therapeutic target.

Furthermore, rs75326924 has previously been associated with CD36 deficiency and reduced CD36 protein expression, further supporting its functional relevance (26). Therefore, unlike many asthma-associated variants located in non-coding regions, rs75326924 is a coding variant with a potential functional link that may directly affect the function of the CD36 protein. However, it remains unclear whether this variant directly alters ligand recognition, receptor trafficking, or lipid uptake in asthma-related cells. Taken together, genetic, airway transcriptomic, cellular, and proteomic data collectively provide human evidence linking CD36 to asthma susceptibility and inflammatory phenotypes. These findings strengthen the biological rationale for investigating CD36 as a candidate immunometabolic regulator in asthma.

## 3 CD36 as an upstream sensor of allergen exposure

### 3.1 Environmental exposure is the initial step in allergic airway inflammation

The development of allergic airway inflammation requires the recognition and processing of inhaled environmental allergens. Prior to the adaptive immune response, allergens must first be detected, endocytosed and presented by airway epithelial cells and antigen-presenting cells (APCs) (27).

Traditionally, this process was thought to rely primarily on pattern recognition receptors, such as TLRs and C-type lectin receptors (28). However, growing evidence suggests that endocytic receptors are also involved in environmental perception. Among these receptors, CD36 occupies a unique position, being capable of recognizing lipid-associated structures commonly found in the air. Because CD36-mediated recognition does not require pre-existing allergen-specific IgE, it is likely to function during the innate phase of allergen recognition, before the establishment of adaptive immune responses.

### 3.2 House dust mite: the strongest evidence for CD36-dependent allergen recognition

House dust mites (HDM) provide the best example of a CD36-mediated allergen recognition mechanism. HDM particles contain not only major allergenic proteins such as Der p1 and Der p2, but also endogenous lipid adjuvants and structures containing phosphatidylcholine (PC), which are capable of interacting with CD36 (29, 30). Respiratory epithelial cells and dendritic cells endocytose these PC-containing particles via CD36-dependent mechanisms, thereby facilitating allergen uptake and processing.

Mechanistically, CD36 acts in synergy with the platelet-activating factor receptor (PAFR), which recognizes PAF-like lipids, oxidized phospholipid mediators with PAF-like activity, present in HDM extracts (23, 31). This interaction allows for the simultaneous occurrence of allergen uptake and inflammatory signaling. Experimental studies have shown that CD36 knockout significantly reduces HDM endocytosis, inhibits Th2 differentiation, decreases the production of specific IgE, and alleviates eosinophil-mediated airway inflammation. Blocking the PC epitope inhibits allergen uptake in wild-type animals but has little effect in CD36-deficient mice, supporting a direct role for CD36 in allergen recognition (23). Interestingly, pediatric asthma patients exhibit lower levels of naturally occurring anti-PC IgM antibodies, providing indirect clinical support for the relevance of the PC-dependent pathway in human disease (32). However, evidence for age-related differences in CD36 expression or function between pediatric and adult asthma remains limited. Whether CD36 has distinct roles during sensitization, established disease, and chronic airway inflammation is also unclear. Similar questions arise across the allergic triad, including allergic rhinitis and atopic dermatitis.

### 3.3 Beyond house dust mite: expanding the spectrum of CD36-recognized allergens

Evidence from HDM serves as the cornerstone for the role of CD36 in allergen recognition, but other data indicates that CD36 may also be involved in responses to a broader range of environmental stimuli. Many airborne allergens contain lipid-binding molecular structures capable of activating scavenger receptors. The cell wall structure of fungi contains various allergenic components (33). For example, CD36 mediates the recognition and uptake of β-glucan from the cell wall of *Aspergillus fumigatus*. In contrast, CD36-deficient mice exhibit impaired uptake of *Aspergillus fumigatus*, reduced CD4^+^ T-cell responses, and exacerbated pulmonary inflammation (34). Environmental pollutants such as particulate matter and ozone can increase oxidative stress in the airways and promote the accumulation of lipid peroxidation products, such as oxidized phospholipids (35, 36). As established ligands for CD36, oxidized phospholipids may therefore promote the development of CD36-dependent inflammatory pathways (37). These observations suggest that the function of CD36 should not be defined as a receptor for a single allergen, but rather as a sensor for conserved lipid-associated danger signals generated during environmental exposure. This broader perspective is crucial because allergic airway diseases are typically not caused by a single allergen; patients are usually continuously exposed to a complex mixture of allergens, microbial products, pollutants, and endogenous damage-associated molecular patterns (DAMPs). CD36 possesses an extremely broad ligand repertoire capable of integrating this diversity.

### 3.4 Cooperation between CD36 and pattern-recognition receptors

Environmental sensing rarely relies on a single receptor. The activation of the innate immune system requires coordinated action among multiple receptor systems. CD36 is a prime example of this principle.

Studies on macrophages and platelets have demonstrated that CD36 forms functional signaling complexes with Toll-like receptors (particularly TLR2 and TLR6) (38, 39). Within these complexes, CD36 facilitates ligand capture and internalization, while downstream inflammatory signals are transmitted via the classical MyD88-dependent pathway, ultimately activating NF-κB and MAPK signaling cascades (13).

As we have mentioned, oxidized phospholipids, microbial products, and environmental particles often act simultaneously on both scavenger receptors and TLRs. Therefore, even if CD36 is not the primary signaling receptor, it may enhance the efficiency and intensity of innate immune activation. Thus, CD36 should not be viewed as an isolated receptor but rather as part of a larger environmental sensing platform capable of amplifying the response to inhaled stimuli.

### 3.5 Linking environmental sensing to T2 immunity

The ultimate outcome of allergen recognition is the initiation of a T2 immune response (40). Based on the evidence presented above, we propose a conceptual framework: exposure to environmental allergens promotes allergen uptake and epithelial cell activation through CD36-mediated sensing, which subsequently triggers the release of alarmin, resulting in the activation of ILC2s and Th2 cells and ultimately inducing T2 airway inflammation. Importantly, this model positions CD36 upstream in the asthma pathway, making it a potential target for biologic therapies. Thus, in theory, modulating CD36 activity could simultaneously influence multiple downstream inflammatory networks. In summary, existing evidence supports the role of CD36 as an environmental sensing receptor involved in the initiation of allergic airway inflammation. However, allergen recognition alone cannot explain the persistence of chronic disease. Sustained inflammation requires long-term cellular activation and metabolic adaptation. Thus, the question arises: how do immune cells maintain their effector functions following initial allergen exposure? This question leads us to one of the fastest-evolving areas in CD36 biology: immunometabolism.

## 4 CD36 links metabolism to T2 immunity

### 4.1 Chronic inflammation requires metabolic adaptation

**Figure 1:**
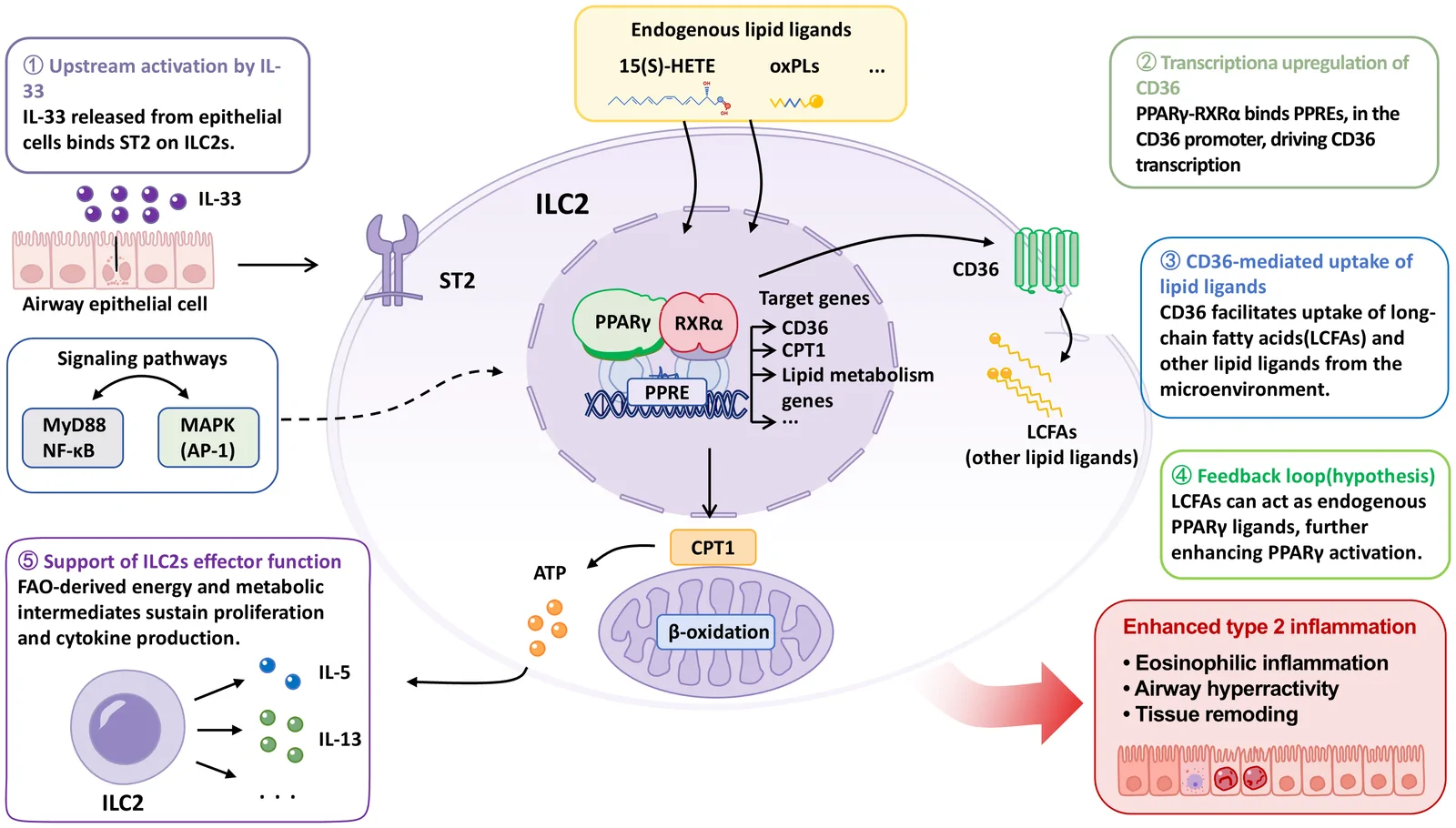
The PPAR-γ-CD36 axis integrates inflammatory signaling and lipid metabolism to sustain ILC2 effector function. Airway epithelial-derived IL-33 activates ST2 signaling in ILC2s, inducing downstream MyD88-NF-κB and MAPK pathways, with current evidence supporting MAPK-AP-1 signaling as an important route promoting PPAR-γ induction. Activated PPAR-γ forms a heterodimer with RXRα and binds PPAR response elements (PPREs) within target genes, including CD36, thereby promoting transcriptional programs involved in lipid uptake and metabolism. CD36 facilitates the uptake of long-chain fatty acids (LCFAs) and other lipid ligands from the inflammatory microenvironment. Imported fatty acids can enter mitochondria through CPT1 and undergo fatty acid oxidation (FAO), generating acetyl-CoA and reducing equivalents that support mitochondrial OXPHOS and ATP production. OXPHOS provides an important source of metabolic support for ILC2 function, including cytokine production. Endogenous lipid mediators such as 15-HETE and oxidized phospholipids may further enhance PPAR-γ activation. A proposed positive-feedback loop in which CD36-mediated lipid uptake reinforces PPAR-γ activity is shown as hypothetical, as direct *in vivo* validation in ILC2s remains lacking. Collectively, the PPAR-γ-CD36 axis links lipid uptake and metabolic adaptation with sustained T2 inflammatory responses in the airway.

The persistence of asthma cannot be explained solely by repeated exposure to allergens. Although environmental cues trigger an immune response, chronic inflammation requires sustained cellular activation, proliferation, survival, and cytokine production (41). These processes place substantial demands on immune cells in terms of energy and biosynthesis. Recent advances in immunometabolism research indicate that immune cell function is closely linked to metabolic status (9). Metabolic pathways not only serve as energy sources but also actively regulate cell fate determination, effector programs, and inflammatory responses. In asthma, metabolic reprogramming has emerged as a key determinant of disease persistence (42). ILC2s, as key effector cells in T2 inflammation, undergo metabolic adaptation upon activation. Although ILC2s can utilize mitochondrial oxidative phosphorylation (OXPHOS) to support cellular energy demands, activated ILC2s also increase lipid uptake and lipid metabolic activity to sustain cytokine production and effector functions. Therefore, lipid metabolism represents an important component of ILC2 metabolic regulation, rather than an exclusive energy source (43). (**Figure 1**).

### 4.2 ILC2s as central drivers of T2 airway inflammation

ILC2s occupy a key position at the interface between the innate and adaptive immune systems. Unlike Th2 cells, ILC2s are activated in an antigen-independent manner and can rapidly respond to epithelial cytokines released in response to tissue injury or allergen exposure, even in mice lacking an adaptive immune system (44). In asthma, this response is primarily driven by IL-33, IL-25, and TSLP (45). Activated ILC2s produce large amounts of IL-5 and IL-13. IL-5 promotes eosinophil differentiation, recruitment, and survival, while IL-13 promotes excessive mucus secretion, goblet cell metaplasia, airway hyperresponsiveness, and tissue remodeling (44). Importantly, sustained activation of ILC2s has been demonstrated in severe asthma and may contribute to residual inflammation despite cytokine-targeted biologic therapies (46). These observations suggest that the mechanisms maintaining ILC2 function may represent a key therapeutic vulnerability.

### 4.3 PPAR-γ establishes a metabolic program for ILC2 function

Among the transcriptional regulators involved in ILC2 biology, the peroxisome proliferator-activated receptor gamma (PPAR-γ) has emerged as a central metabolic regulator. PPAR-γ is highly expressed in activated ILC2s and is involved in regulating lipid uptake, fatty acid oxidation, and mitochondrial metabolism (47). More importantly, PPAR-γ is essential for ILC2 activation; pharmacological inhibition or deletion of the PPAR-γ gene significantly reduces ILC2 proliferation, suppresses IL-5 and IL-13 production, and markedly alleviates allergic airway inflammation in mouse models (19). Additionally, Xiao et al. demonstrated that PPAR-γ directly regulates the expression of the IL-33 receptor ST2, thereby linking metabolic programming to alarmin responsiveness (48). These findings suggest that metabolic regulation is not a secondary consequence of immune activation but rather an integral component of the ILC2 effector program.

### 4.4 The PPAR-γ-CD36 axis supports the metabolic fitness of ILC2s

A central question in ILC2 biology is how these cells maintain their effector function during chronic T2 inflammation. Given the substantial energy requirements associated with sustained cytokine production, metabolic adaptation is a prerequisite for the long-term activation of ILC2s.

CD36 is widely considered a downstream transcriptional target of PPAR-γ (14). Activation of PPAR-γ promotes the transcriptional upregulation of CD36, enhancing the uptake of long-chain fatty acids (LCFAs). These lipids subsequently enter the mitochondrial β-oxidation pathway, producing the ATP and metabolic intermediates required to support cell survival and cytokine production (19).

For this reason, the PPAR-γ-CD36 axis provides a possible mechanistic framework linking epithelial-derived danger signals to persistent T2 inflammation. Following epithelial injury, the release of IL-33 activates ILC2s and initiates metabolic programs that support their effector functions (11). In this process, PPAR-γ-dependent upregulation of CD36 may promote LCFA uptake and downstream β-oxidation, thereby facilitating the metabolic adaptations required for sustained production of IL-5 and IL-13 (11, 19). This pathway does not constitute a simple linear signaling cascade but is increasingly viewed as part of a broader immune metabolic network that drives chronic T2 inflammatory responses.

Recent studies have further emphasized the importance of extracellular fatty acids as metabolic substrates for ILC2 activation (11, 49). Unlike highly glycolytic effector T cells, ILC2s rely more heavily on fatty acid metabolism to maintain their effector function. Long-chain fatty acids taken up via transport systems—including CD36—support mitochondrial respiration and promote sustained cytokine production (12, 50). Thus, CD36 not only facilitates lipid uptake but also contributes to the metabolic adaptations required for sustained ILC2 activation.

Experimental data indicate that pharmacological or genetic inhibition of PPAR-γ reduces CD36 expression and impairs fatty acid uptake, while simultaneously decreasing cytokine production in activated ILC2s (19). Transcriptomic analyses of human and mouse ILC2 populations also indicate that lipid metabolic pathways—including the CD36-related gene network—are enriched during ILC2 activation (50). Although direct evidence establishing the IL-33-PPAR-γ-CD36 axis in human asthma remains limited, the available data generally support the role of CD36-dependent lipid metabolism in maintaining the ILC2 effector response.

## 5 CD36 as an amplifier of T2 inflammation

### 5.1 Beyond allergen recognition and metabolism

CD36 is involved in both the initiation of allergic reactions and the metabolic maintenance of T2 immunity. New evidence suggests that CD36 may also be involved in the amplification of inflammatory signals (51, 52). Unlike receptors that function only at a specific stage of immune activation, CD36 is widely expressed on various immune cells and interacts with different signaling pathways (14). This broad biological function suggests that CD36 may act as a molecular amplifier, linking environmental sensing, innate immune activation, and the persistence of chronic inflammation.

### 5.2 CD36 and pattern-recognition receptor signaling

As described in section 3.4. CD36 forms signaling complexes with TLR2 and TLR6 to facilitate the recognition of oxidized phospholipids, microbial lipoproteins, and endogenous DAMPs. Ligand binding promotes the recruitment of Src family kinases and activates downstream NF-κB and MAPK signaling pathways, thereby enhancing the production of pro-inflammatory mediators (13, 53).

Oxidative stress is a hallmark of the inflamed airway and leads to the accumulation of oxidized lipids and phospholipids in the local tissue microenvironment (54). Among these, several oxidized phosphatidylcholines (such as POVPC, PGPC, KOdiA-PC, and members of the oxPCCD36 family) have been shown to be high-affinity endogenous ligands for CD36 in experimental systems (55, 56). These oxidized lipids accumulate under conditions of oxidative damage and inflammation and can activate CD36-dependent signaling pathways (52). Although most evidence comes from studies of macrophages and vascular inflammation models, the presence of oxidative stress and oxidized phospholipids in the airways of asthma patients suggests that similar mechanisms may also be involved in inflammatory signaling within the airway microenvironment. From this perspective, CD36 continuously generates endogenous danger signals by regulating innate immune signaling, amplifying the local inflammatory network.

### 5.3 CD36 and macrophage functional states

Macrophages represent another important cellular environment in which CD36 may influence the pathogenesis of asthma. As abundant innate immune cells in the airways, macrophages play a crucial role in tissue homeostasis, inflammation regulation, and the clearance of cellular debris (57). Their remarkable functional plasticity enables them to exhibit different states of activation in response to local environmental signals. In allergic respiratory diseases, macrophages with distinct activation profiles are associated with eosinophilic inflammation, tissue remodeling, and excessive mucus secretion (58).

CD36 is highly expressed in macrophages and regulates multiple aspects of macrophage biology, including lipid uptake, peripheral phagocytosis, and innate immune signaling (59). Under specific experimental conditions, CD36-dependent lipid processing may influence macrophage activation pathways and promote phenotypes associated with tissue repair and T2 immunity (60). Since activated alternative macrophages can produce mediators such as CCL17, CCL22, TGF-β, and arginase-1, CD36-dependent metabolic pathways may indirectly regulate downstream T2 inflammatory responses (61, 62). However, much of the mechanistic evidence for macrophage-associated CD36 derives from pulmonary fibrosis and other non-asthmatic models, where CD36 has been implicated in oxidized lipid handling and profibrotic macrophage responses (14). Whether these mechanisms are relevant to T2 inflammation in asthma remains unclear. Direct evidence linking macrophage-expressed CD36 to allergic airway inflammation is therefore limited, warranting further investigation of its disease-specific role in asthma.

### 5.4 Potential interactions between CD36 and eosinophilic inflammation

Eosinophils are the hallmark effector cells of T2 asthma and cause airway damage by releasing cytotoxic granule proteins, lipid mediators, and cytokines (63). Human genetic studies have shown that CD36 is associated with eosinophilic inflammation. Individuals carrying the protective rs75326924 variant have lower peripheral blood eosinophil counts than non-carriers, suggesting that alterations in CD36 function may influence eosinophil-related signaling pathways (20).

Regardless of the exact mechanism, these observations support the notion that CD36’s influence extends beyond the inflammatory networks of a single cell type. T2 airway inflammation involves a bidirectional feedback loop between ILC2s and eosinophils: IL-5 produced by ILC2s promotes eosinophil recruitment, while activated eosinophils enhance the effector function of ILC2s (64). Eosinophil-derived cysteinyl leukotrienes (LTC_4_, LTD_4_, and LTE_4_) rapidly induce IL-5 and IL-13 production by ILC2s, predominantly through CysLT1R. This response is further potentiated by prostaglandin D_2_ (PGD_2_) and epithelial-derived alarmins, including IL-25, IL-33, and thymic stromal lymphopoietin (TSLP), leading to robust amplification of ILC2 activation (65, 66), their hydrophobic lipid backbone may overlap with CD36-mediated lipid uptake pathways (66).

In other myeloid cell systems, CD36 can act in concert with receptors such as PAFR to participate in lipid-related inflammatory signaling (67, 68). However, it remains unclear whether similar mechanisms exist in ILC2s. Clinically, disease exacerbation that persists despite anti-IL-5 therapy may reflect ILC2s with elevated CD36 expression or enhanced fatty acid oxidation, thereby maintaining a state of activation independent of eosinophils (69–71). In summary, these findings support a more refined model: CD36 integrates lipid-mediated signaling within the eosinophil-ILC2 axis, thereby amplifying T2 inflammatory responses.

Taken together, CD36 may enhance T2 inflammatory responses through multiple, partially overlapping mechanisms. CD36 plays a system-wide regulatory role at the intersection of metabolism, innate immunity, and chronic inflammation. This perspective helps explain why genetic variants affecting CD36 can still influence disease susceptibility.

## 6 CD36 and airway remodeling: emerging evidence beyond inflammation

### 6.1 Airway remodeling as a determinant of disease progression

While airway inflammation remains a key feature of asthma, structural changes in the airway wall are the fundamental determinants of the disease’s long-term progression (72). These include: subepithelial fibrosis; goblet cell hyperplasia; airway smooth muscle hypertrophy; extracellular matrix deposition; and vascular remodeling.

These changes lead to airflow limitation, decreased lung function, and, in some patients, partially irreversible airway narrowing. Traditionally, remodeling has been viewed as a downstream consequence of chronic inflammation. However, CD36 may act as a key regulatory factor linking inflammation, lipid metabolism, and structural remodeling based on indirect evidence.

Although direct evidence linking CD36 to airway remodeling in asthma remains limited, studies in pulmonary fibrosis have implicated CD36 is involved in multiple fibrotic processes such as lipid metabolism, macrophage activation, TGF-β signaling, and extracellular matrix remodeling (73, 74). Thus, a similar CD36-dependent mechanism that may promote structural remodeling in asthma provides the conceptual basis.

### 6.2 CD36 and TGF-β activation: a potential fibrotic pathway

Thrombospondin-1 (TSP-1) is a matricellular glycoprotein known for its ability to activate latent transforming growth factor-β1 (L-TGF-β1) (75). Studies have shown that TSP-1 anchors latent L-TGF-β1 to the surface of alveolar macrophages via CD36, thereby promoting its subsequent proteolytic activation (76). Consistent with this mechanism, *in vitro* systems and bleomycin-induced pulmonary fibrosis models have shown that disruption of the TSP-1-CD36 interaction reduces L-TGF-β1 activation and attenuates collagen deposition (77).

Furthermore, CD36-mediated recognition of oxidized phospholipids has been shown to activate Src family kinases (including Lyn) and enhance pro-fibrotic signaling in macrophages. In experimental models, deletion of the CD36 gene partially protects against fibrosis (74). In summary, these findings suggest that CD36 may be a potential regulator of TGF-β-dependent tissue remodeling in a fibrotic environment. Although airway remodeling in asthma differs in nature from interstitial fibrosis, both involve extracellular matrix deposition and TGF-β-driven structural changes. Therefore, findings from fibrosis models suggest that CD36-dependent signaling pathways may also be involved in airway remodeling. However, there is currently a lack of direct evidence supporting the existence of a functional TSP-1/oxidized phospholipid-CD36-TGF-β axis in the airways of asthma patients, and its relevance to human disease remains unclear.

### 6.3 CD36 and vascular remodeling

Vascular remodeling is a characteristic yet often overlooked aspect of chronic asthma. Increased vascular density, endothelial cell activation, and enhanced vascular permeability result in thickening of the airway wall and facilitate the recruitment of inflammatory cells to the airway microenvironment (78). Evidence from vascular biology reveals that CD36 plays a complex and context-dependent role in angiogenesis. By interacting with TSP-1, CD36 inhibits endothelial cell proliferation and promotes apoptosis, thereby exerting anti-angiogenic effects (79, 80). Conversely, under specific physiological conditions, CD36-mediated fatty acid uptake may support endothelial cell metabolism and tissue repair (81). The former used TSP-1, while the latter used oleic acid (OA). This indicates that CD36 may initiate distinct signaling pathways by binding to different ligands.

These seemingly contradictory findings show that CD36 likely functions as a regulator of vascular homeostasis rather than a simple pro- or anti-angiogenic molecule. Interestingly, individuals carrying the protective rs75326924 variant allele exhibit lower circulating VEGFA levels (20), suggesting that altered vascular responses may help explain the protective phenotype associated with reduced CD36 function. Although a causal relationship has not yet been established, this observation provides indirect support for further investigation of CD36-mediated vascular pathways in asthma.

### 6.4 The role of CD36 at the interface between inflammation and structural changes

First, CD36 is involved in the recognition and clearance of apoptotic cells (peripheral phagocytosis), a process that is critical for tissue homeostasis and the resolution of inflammation. Impaired clearance of apoptotic cells may lead to persistent chronic inflammation and abnormal tissue repair (82). Second, CD36 influences macrophage functional states and metabolic adaptation, both of which affect the wound healing response and extracellular matrix renewal (59). Third, CD36-dependent signaling pathways intersect with those regulating TGF-β activation, angiogenesis, oxidative stress, and lipid metabolism. These observations suggest that CD36 may act as a molecular bridge linking chronic inflammation to structural adaptations in the airway wall.

## 7 Translational opportunities: CD36 as a biomarker and therapeutic target

**Figure 2:**
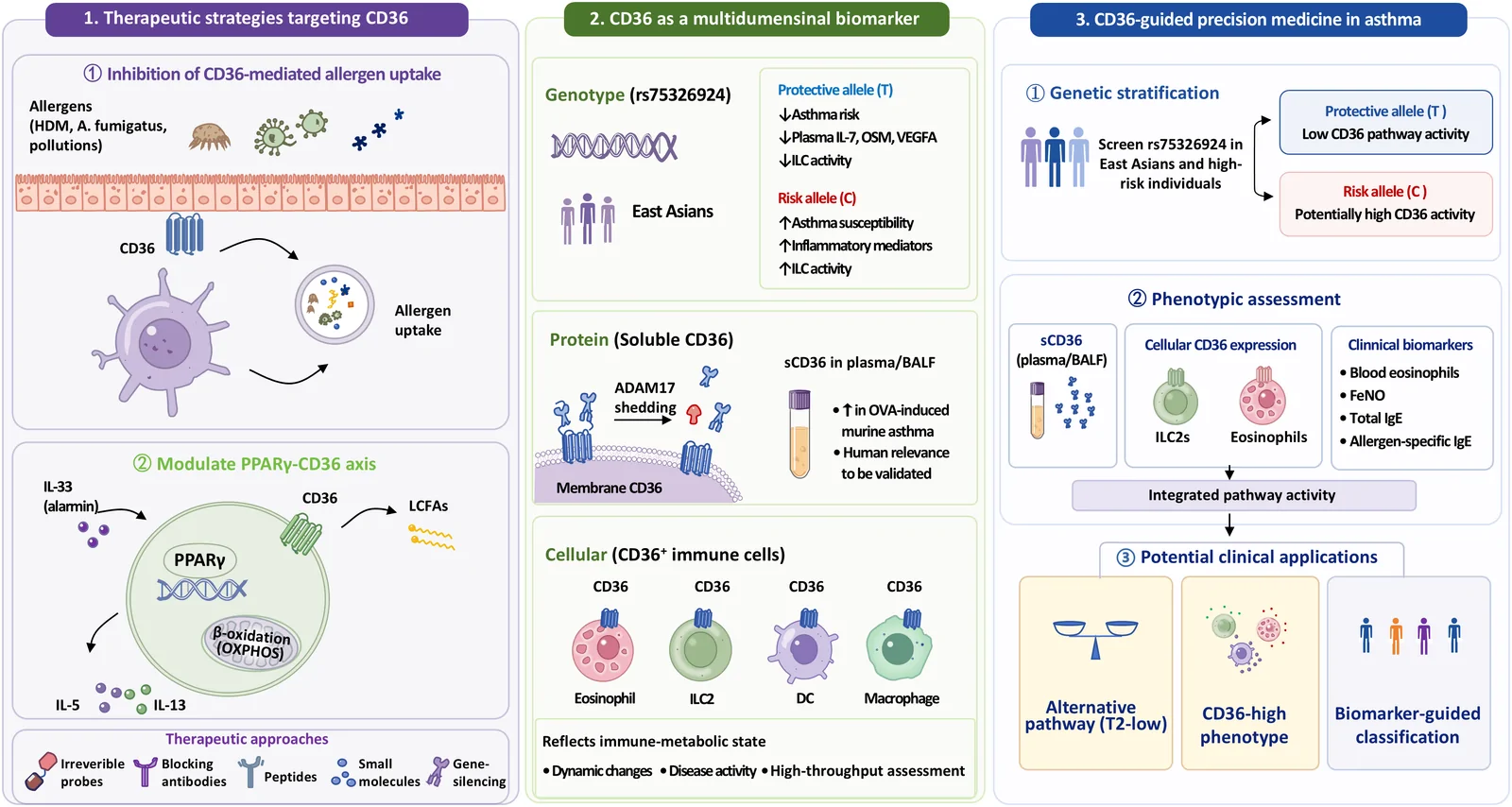
Toward CD36-guided precision medicine in asthma: a conceptual translational framework integrating biomarkers and therapeutic opportunities. The figure illustrates a three-layer framework linking CD36-associated genetic variants, multi-level biomarker profiles, and potential translational applications in asthma. The left panel represents potential options for the treatment of asthma by targeting CD36. The middle panel integrates soluble (sCD36 in plasma and BALF), cellular (ILC2s and eosinophils), and established clinical biomarkers (blood eosinophils, FeNO, total IgE, allergen-specific IgE) on a unified CD36-related pathway activity axis reflecting disease heterogeneity. The right panel Outlines potential translational implications, including biomarker-based patient classification and identification of CD36 high expression and T2-low inflammatory backgrounds.

The translation of CD36 biology into clinical strategies represents a frontier in airway T2 inflammation research. There are two main treatment approaches: blocking allergen uptake and modulating immune-metabolic programs. Simultaneously, CD36’s accessibility across multi-omics layers presents potential as a precision medicine biomarker (**Figure 2**).

### 7.1 Strategy 1: inhibition of CD36-mediated allergen uptake

Targeting the earliest step of allergen recognition and internalization is conceptually attractive for preventing sensitization and acute exacerbations. *In vitro* studies show that SSO covalently modifies Lys164 and Lys166 in CD36, irreversibly blocking the lipid-binding pocket and preventing uptake of oxidized lipids and allergens (23, 83). However, due to its irreversible mechanism and interference with systemic lipid metabolism, SSO is primarily a mechanistic tool rather than a clinically viable agent.

Recent efforts have focused on developing more selective approaches to modulate CD36 activity, including inhibitory peptides and antibody-based strategies. The CD36-selective peptide EP80317 has been shown to exert CD36-dependent protective effects in experimental models of atherosclerosis and cardiac injury (84). More recently, a fully human anti-CD36 single-chain variable fragment (scFv), termed D11, was identified and shown to bind CD36 and inhibit ligand uptake in macrophage and hepatocyte models. D11 reduced oxLDL-induced foam cell formation and lipid accumulation, providing proof-of-concept that CD36-mediated lipid sensing can be therapeutically modulated (85). However, whether antibody-based or peptide-based CD36 inhibition can influence allergic airway inflammation remains unknown, and further studies are required to determine the therapeutic relevance of CD36 targeting in asthma.

### 7.2 Strategy 2: modulation of the PPAR-γ-CD36 metabolic axis

In established chronic T2 inflammation, allergen uptake inhibition alone may be insufficient. Targeting effector cell metabolism, particularly ILC2s, represents an alternative. ILC2 effector functions are tightly coupled to PPAR-γ-driven lipid metabolic programs. Global modulation of PPAR-γ is challenging; antagonists such as GW9662 may induce systemic metabolic dysregulation, whereas thiazolidinediones exhibit anti-inflammatory effects but carry risks of weight gain and fluid retention (86).

Targeting downstream CD36 offers a narrower, more specific intervention point. By limiting ILC2 uptake of long-chain fatty acids, CD36 modulation could potentially reduce metabolic support for cytokine production without broadly perturbing systemic glucose or lipid homeostasis.

### 7.3 CD36 as a multidimensional biomarker

Genetic variations, tissue distribution, and induced expression of CD36 point to its potential as a biomarker at multiple levels:

Genotype-level stratification: The rs75326924 missense variant provides a natural risk stratification in East Asians, with the protective allele associated with lower asthma incidence, reduced plasma IL-7, OSM, and VEGFA, and altered ILC2 activity. Genotype-based testing is low-cost, non-invasive, and stable throughout life.

Soluble CD36 (sCD36): CD36 ectodomain shedding via ADAM17 generates measurable sCD36 in plasma and BALF (86). In OVA-induced murine asthma, BALF sCD36 increases significantly. Its relevance in humans remains to be validated.

Cellular phenotyping: Surface CD36 on circulating immune cells may reflect immune-metabolic state. While peripheral ILC2s are rare, advances in high-parameter flow cytometry make dynamic monitoring feasible (87). Such measurements could serve as early pharmacodynamic indicators in exploratory trials.

Toward CD36-guided Precision Medicine: Taken together, CD36-associated readouts may offer complementary information across genetic, molecular and cellular levels. Although their clinical utility remains unproven, integration of these measurements could ultimately contribute to a more refined understanding of asthma heterogeneity and support future precision medicine approaches. Prospective studies will be required to determine whether CD36-associated biomarkers provide predictive value beyond established clinical and inflammatory markers.

### 7.4 Lessons from human genetics

Human genetics provides one of the strongest arguments supporting further investigation of CD36 as a therapeutic target. The protective rs75326924 variant represents a naturally occurring model of partial CD36 dysfunction. Individuals carrying this variant exhibit reduced asthma susceptibility without evidence of severe developmental abnormalities. The observation is particularly important because therapeutic targets supported by human genetic evidence frequently demonstrate higher probabilities of successful clinical translation.

However, important caveats must be considered. Genetic variants typically exert lifelong and often partial effects on protein function, whereas pharmacological interventions may induce more profound and temporally distinct alterations. Consequently, therapeutic inhibition of CD36 cannot be assumed to replicate the biological consequences observed in variant carriers.

### 7.5 Challenges and safety considerations

Despite its biological appeal, CD36 presents several challenges as a therapeutic target. First, CD36 exhibits broad tissue distribution and participates in diverse physiological processes extending well beyond immunity. Second, its biological effects are highly context dependent. In some settings CD36 promotes inflammation, whereas in others it contributes to tissue repair and homeostasis. Third, long-term suppression of CD36-dependent pathways could potentially impair host defense, metabolic adaptation, or tissue repair mechanisms. These considerations highlight the importance of identifying disease-relevant cellular compartments and defining therapeutic windows before clinical translation.

## 8 Conclusions

Recent advances in human genetics, immunology, and immunometabolism have substantially expanded our understanding of CD36 beyond its traditional role as a lipid transporter.

Accumulating evidence suggests that CD36 participates in multiple stages of asthma pathogenesis, ranging from environmental allergen sensing and innate immune activation to metabolic regulation of T2 immunity and potentially structural airway remodeling. A particularly important emerging concept is that CD36 functions as an integrative signaling hub linking environmental exposures with cellular metabolic adaptation. Through regulation of allergen recognition, inflammatory amplification, and ILC2 metabolic fitness, CD36 may influence both the initiation and persistence of allergic airway inflammation.

Importantly, the discovery of the protective rs75326924 variant provides strong human genetic support for disease relevance and distinguishes CD36 from many candidate pathways supported solely by experimental observations. Nevertheless, significant uncertainties remain regarding cell-specific functions, interactions with established biologic pathways, and the feasibility of therapeutic targeting.

Collectively, current evidence suggests that CD36 may function as a candidate immunometabolic checkpoint at the intersection of environmental sensing, metabolism, and chronic airway inflammation. Continued integration of human genetics, systems immunology, and translational research will be essential to determine whether modulation of CD36 can ultimately contribute to precision management of asthma.
